# User interface design in mobile learning applications: Developing and evaluating a questionnaire for measuring learners' extraneous cognitive load

**DOI:** 10.1016/j.heliyon.2024.e37494

**Published:** 2024-09-05

**Authors:** Masyura Ahmad Faudzi, Zaihisma Che Cob, Masitah Ghazali, Ridha Omar, Sharul Azim Sharudin

**Affiliations:** a Institute of Informatics and Computing in Energy, Universiti Tenaga Nasional, Malaysia; b Malaysia-Japan International Institute of Technology, Universiti Teknologi Malaysia, Malaysia

**Keywords:** Mobile learning, Human-computer interface, Distance education and online learning

## Abstract

Mobile learning is increasingly popular due to its flexibility in timing and location. However, challenges such as small screen sizes and poor user interface design can elevate learners' cognitive load, especially extraneous cognitive load, which hinders learning. Extraneous cognitive load, stemming from user interface design complexity, must be minimized to enhance learning focus. Currently, there is no dedicated instrument for measuring extraneous cognitive load specific to mobile learning user interface design. This study aims to develop and evaluate a subjective instrument for measuring extraneous cognitive load caused by user interface design in mobile learning applications. Two sets of experiments were conducted: pretesting to establish the instrument's foundation with a small participant group, followed by pilot experiments to validate the instruments and refine experimental procedures. The NASA-TLX score was used to analyze the relationship between overall cognitive load and extraneous load across various user interface criteria. Understanding these relationships can guide user interface improvements to reduce extraneous cognitive load. Challenges encountered during pretesting and pilot experiments included participant fatigue, scale reliability issues, and incomplete data collection. To enhance reliability, adjustments were made: tasks were reduced, the scale was expanded from a 4-point to a 10-point format, and facilitators thoroughly verified data before participants concluded sessions. Creating a tool to measure how user interface design impacts users' extraneous load is important because it is the UI design, not the mobile app's content that affects extraneous load. However, general methods for measuring cognitive load may not accurately identify problems with user interface design. Therefore, an extraneous load-based method is needed. This will also eventually improve usability.

## 1 Introduction

Mobile learning is defined as a learning process, in terms of pedagogy and education [1], that takes place through the learner's own personal mobile devices [2,3]. Using the mobile technology, mobile learning can be accessed from any location at any time [4,5]. However, mobile learning is less convenient than desktop learning [6], where one of the main problems is the small screen size [[7], [8], [9]]. The small screen size makes typing and searching difficult on mobile devices [10,11]. This can increase the cognitive load on the learner and lead to poor learning performance [12].

Unnecessary burden on learners' cognitive systems, notably the extraneous cognitive load, might be added if a poorly designed user interface (UI) is implemented on a mobile learning application (MLA) [[13], [14], [15], [16], [17]]. Bad user interface design (UID) requires learners to relearn how to utilize mobile devices [18]. It is also important to note that not all learners have the required digital skills to confidently explore and interact with mobile learning technology [19]. Fortunately, the extraneous cognitive load (ECL) can be minimized by having a good UID [20].

Good UID are interfaces that were designed based on UID guidelines/frameworks [21,22]. However, the current available UID guidelines/frameworks for learning, such as Mayer's Multimedia Principles [23] or Principles of E-Learning [24], were introduced for desktop application developments, and not for MLA developments. There has been little to no research focusing on developments guideline/framework for MLA [25]. Therefore, it is important to develop a specific UID guideline/framework for MLA. The focus of this guideline/framework is to minimize ECL caused by UID of an MLA.

It is therefore vital to concentrate on measuring the ECL caused by the UID of an MLA. A UID-specific tool helps identify which design elements most impact ECL, offering clear insights for making effective design changes that improve usability. Improving mobile app usability requires a mix of user-centered design principles, thorough usability assessments, and a good understanding of cognitive load.

There were instruments that were developed to specifically measure ECL, such as by Leppink et al. [26,27] and Cierniak et al. [28], where these instruments measure the ECL from the perspective of the clarity and effectiveness of the instructions and language used [27], and difficulties when using the learning material [28]. These instruments were not designed to measure the ECL caused by UID of an MLA. On the other hand, NASA-TLX [29] was designed primarily for evaluating cognitive load caused by UID [30], however, it cannot explicitly measure ECL. Besides understanding the needs, capabilities, and limitations of a user, it is also important to understand how ECL can be minimized through UID.

The purpose of this study is to develop and evaluate an instrument that can be used to measure ECL caused by UID of an MLA. By understanding the UID factors that contributed to ECL, MLA designers, developers and teachers can identify ways to have better UID for MLA. In order to develop the instrument, the research questions (RQ) can be divided into two parts, which are:

RQ1 What are the constructs required to evaluate the UID factors of an MLA, that increase the ECL of a learner?

RQ2 What is the suitable experimental setup to evaluate the UID factors of an MLA, that increase the ECL of a mobile learner?

The remaining part of this article is structured as follows: Section 2 discusses the background and related works on mobile learning, cognitive load, and its measurement methods. This is then followed by Section 3, which presents the methodology for the development of the instrument to measure learner's ECL on UID for MLA, along with pretesting and pilot experiment that evaluates the questionnaire. Section 4 presents the results, discussions and issues faced during the implementation of the pretesting and pilot experiment. Finally, Section 5 provides the conclusion, limitations and suggestions for future work.

## 2 Related work and literature

Mobile learning is a learning technique that allows students to access learning materials at any time and from any location by using mobile devices such as phones and tablets. It has served as a supplement to regular in-class instruction before the arrival of Covid-19 in 2020 [31]. During the pandemic, most students around the world were compelled to rely more on online learning to reduce interaction. As ownership of personal computers are not affordable options for the majority, mobile devices have become the natural online learning gadgets for many learners [32].

Among the advantages of mobile learning is that it can help to increase learners’ participation and achievement [13]. Learning through mobile devices allows the learners to control the pace of their learning, change learning context from formal to informal, and change from learning individually to learning socially [33].

Despite these advantages, most of the mobile learners goes back to using desktop machines due to the inconvenience of small mobile gadgets’ screen sizes [34]. The small screen size makes it more difficult to use keypads and buttons on mobile devices [8]. It also leads to problems with readability, e.g., small font size, dense text and small text on complex images [35]. A small screen requires excessive mental resources and focuses on reading and researching necessary information [35]. Small screen comes with smaller images, which is also a challenge for learners [36]. Another impact of the small screen is that too much information needs to be presented on a single interface [12]. In another word, the small screen size reduces the learning experience, the effectiveness of the learning process while taking away the delightful and engaging experience of mobile learning [7,8].

Besides the small screen size, the MLA must also be free from design issues to ensure effective learner engagement [37]. Thus, the UI design of an MLA is crucial for learning adoption and effectiveness [38] Recent studies indicate that Generative AI (GenAI) can enhance creativity and speed-up UI design, but it also risks promoting surface learning and fostering over-reliance [39]. The ease of generating high-quality solutions using GenAI, might lead designers to refine existing ideas instead of exploring new alternatives [40].

This is where UID guidelines/frameworks take up the guidelines for designing UID that are free from errors, which will lead to high degree of learning adoption and effectiveness [21,22]. However, the number of UID guidelines/frameworks specifically for MLA is limited [36]. Issues such as the lack of tactile feedback, the constrained screen size, and the high demands on visual attention were not the concern of UID guidelines/frameworks for desktop application (app) [41,42]. Additionally, MLA is meant to be used for teaching and learning, therefore the app cannot simply be developed by following the same guidelines as general mobile apps, such as iOS - Human Interface Guidelines [43] or Google's Material Design Guidelines [44].

A systematic literature review has been conducted to understand the guideline/framework that are currently being applied when developing MLA [25]. In the study, it has been identified that Nielsen's Heuristics are the most used guideline when developing MLAs, followed by Mayer's Multimedia Principles. However, several researchers discovered significant difficulties with Nielsen's Heuristics, deeming it unsuitable for mobile apps and allowing some usability issues to go undiscovered [[45], [46], [47]]. Mayer's Principles are also facing some issues with the redundancy principle, when it is unsuitable when learning is “on the go” or implemented in a noisy environment [36].

As mobile learning may become one of the methods of teaching and learning in the future, it is critical to have UID guidelines for MLA [32]. UID can significantly influence a learner's ability to grasp knowledge easily or find the learning process challenging [19,32]. Galitz [21] and Wilson et al. [22] have established that failure to follow UID guidelines results in poor UI. A well-designed UI can contribute significantly to a positive user experience, leading to increased engagement and motivation in using MLA [48,49].

Bad UI design places a high demand on visual attention, leading to higher cognitive load [41,42], which lead to higher cognitive load. Cognitive Load Theory [38] introduced three different types of loads, which are the intrinsic cognitive load (ICL), extraneous cognitive load (ECL) and germane cognitive load (GCL). ICL is the complexity of the learning task and ECL is caused by the way the information is being presented [38], and it is the load that can be reduced through the design of the leaning materials [50]. GCL is defined as the activities that facilitate learning and contribute to knowledge transfer [51].

The main objective of a learning system is to minimize the ECL [52,53] to enable the mental resources to be used for GCL [51]. In another word, the UID should allow learners to focus on the learning tasks rather than trying to understand the navigation of the app [19]. Extraneous load pertains to how a task is represented and interpreted by the user and can be influenced by design choices. It represents the connection between the visual presentation of a task and the resulting workload [54].

There are two methods for measuring cognitive load: objective and subjective [48]. Objective measurement method includes eye tracking, dual-task paradigm, face expression analysis and bio trace. Despite demanding a complicated experiment setup, the objective methods are able to provide a more valid and reliable result [54].

Subjective methods are methods that include surveys and questionnaires. Surveys and questionnaires are examples of subjective approaches. Cognitive Load Questionnaires by Pass and Merrienboer [49], Cognitive Load Questionnaires by Sweller [33,34], a combination of both Cognitive Load Questionnaires by Sweller and Pass and Merrienboer [50,51], Cognitive Self-Reported Scale by Badawi [14] and NASA Task Load Index (NASA-TLX) [52] are some of the most commonly used subjective methods.

According to their review, Mutlu-Bayraktar et al. mentioned that although objective methods provide more valid and reliable ways to measure cognitive load, subjective methods are more often used when measuring cognitive load [55]. Subjective measures are advantageous for cognitive load research because they do not require a complex experimental setup and can be easily implemented and repeatedly used in various research designs [56]. Subjective methods can also offer valuable insights into extraneous cognitive load by capturing user experience, while objective methods tend to provide a more direct but less specific measure of overall cognitive load [53,57].

Several subjective methods were designed primarily for evaluating cognitive load caused by UID. This includes NASA-TLX [29,30] and Questionnaire for User Interaction Satisfaction (QUIS) [58]. However, both methods cannot explicitly measure ECL.

Subjective measures that can specifically measure ECL, were developed by Cierniak et al. [28] and Leppink et al. [26,27]. Although they were developed in 2009 and 2013, respectively, they are still the most frequently used method [55]. These instruments focus on the extraneous effects caused by the complexity, clarity, effectiveness of the instruction, and the difficulty of understanding the material used for learning the subject, but not on the UID of the system. In this case, the instruments developed were either evaluating ECL caused by the learning materials (instrument by Cierniak [28] and Leppink [26,27]) or evaluating cognitive load as a whole caused by the UID (NASA-TLX [29] or QUIS [58]), but not evaluating ECL caused by UID.

It is crucial to have a specific tool for measuring extraneous load in UID because this type of load arises from the interface's design rather than the inherent complexity of the learning material (intrinsic load) [12,59]. General cognitive load measurement methods might not be able to distinguish between MLA's UID and learning content, thus making it difficult to determine if UID is the cause of high ECL [60].

**Fig. 1:**
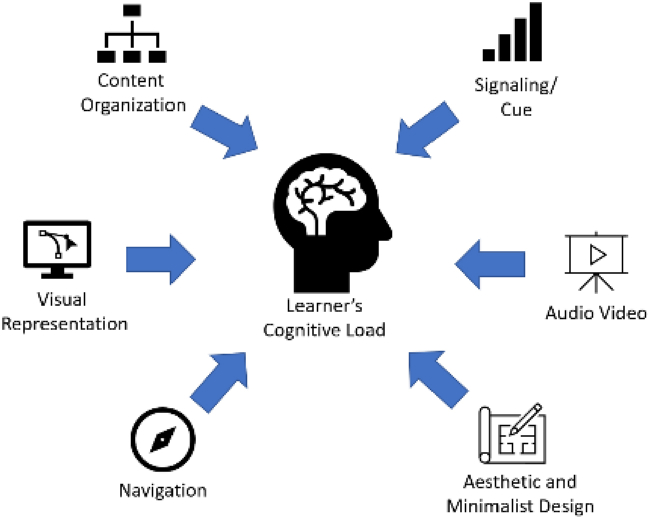
UID impact on learner's cognitive load (adapted from Faudzi et al.’s work [59]).

A study by Faudzi et al. [59] identified six UID criteria that specifically increase ECL, as illustrated in Fig. 1. These criteria are content organization, navigation, signaling/cue, audio/video, aesthetic and minimalist design and visual representation. Among the issues faced by the MLA users includes inappropriate layout structure, confusing navigation, complexity of the graphics and complicated screen design.

Understanding the impact of these UID criteria on a mobile learner's cognitive load requires an instrument that evaluates the criticality of the UID on the learner's ECL. High ECL will affect the performance of the learners' memory, which will then lead to a drop in the task performance [61]. The results of the measurement can be used as reference by designers, developers, and teachers to understand and predict the level of cognitive load in the early design phase [62].

## 3 Methodology

**Fig. 2:**
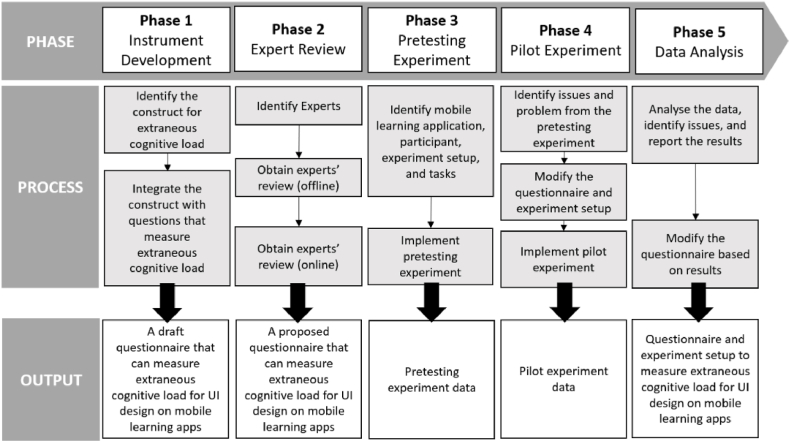
Flow of the development and evaluation of the questionnaire.

The process of developing and evaluating the questionnaire to measure the ECL caused by UID of an MLA is divided into five phases: instrument development, expert review, pretesting, pilot experiment, and data analysis. The flow can be seen in Fig. 2.

**Fig. 3:**
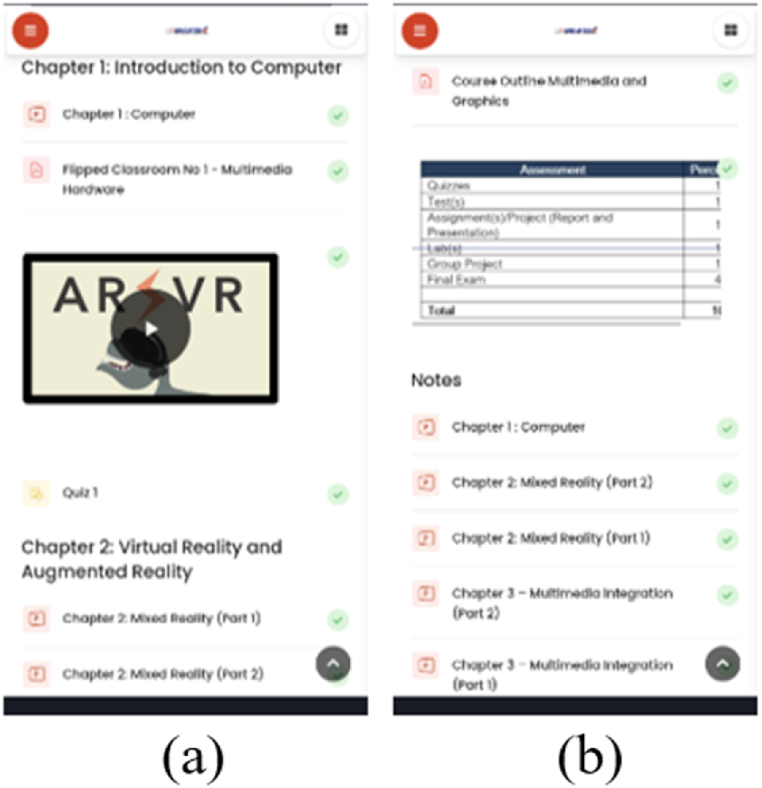
(a) Layout A for task 1 (b) layout B for task 1.

### 3.1 Phase 1: Instrument development

#### 3.1.1 Identification of construct

As demonstrated in earlier work, UID issues that can increase learner's ECL has been identified and categorized based on their criteria [59]. Based on the UID issues obtained, constructs for each issue have been identified and are shown in Table 1.

**Table 1:** Identified construct based on UID issues.

UID Criteria for Mobile Learning App	UID Issues	Identified construct
Content Organization	Inappropriate layout structure	Layout structure
Navigation	Unstructured/confusing navigation	Links
Signaling/Cue	No signal/cue	Signal/cue in terms of color difference, highlights and labels
Audio/video	Animation with narratives and on-screen text	Redundancy of animation and narration
Aesthetic and Minimalist Design	Complicated and difficult to understand format	Page design
Visual Representation	Small/inappropriate font size	Font size, font style
	Complexity of graphics	Complexity of the graphic structure

#### 3.1.2 Integration of constructs with questionnaire

In this phase, instruments developed by Leppink et al. [26,27], and Cierniak et al. [28] were used as reference as their instruments feature questions that are specifically developed for measuring the ECL. While originally proposed more than 10 years ago, the instruments described have continued to be significant. Recent studies have reaffirmed its utility, demonstrating that the foundational principles it established are still critical for current research [63,64]. These instruments were chosen as they were able to measure ECL, specifically.

Table 2 depicts the questions and statements from those articles [[26], [27], [28]] that measure ECL, with the integration of the questions with the construct identified in Table 1. This integration was developed based on conceptual framework for mobile learning UID for improving learner's ECL [59]. The integration between measuring ECL and UID criteria is important as it can show the relationship between ECL and UID criteria. The proposed instrument used a 5-point Likert scale ranging from Strongly Disagree to Strongly Agree for measurement as it is the most used scale in education research [65].

**Table 2:** Questions integration based on Leppink et al., 2013 and Cierniak et al., 2009.

ECL questionnaire [26,27]	ECL questionnaire [28]	Integrated questions based on constructs
The instructions and/or explanations during the activity were very unclear. The instructions and/or explanations were, in terms of learning, very ineffective. The instructions and/or explanations were full of unclear language.	How difficult was it for you to learn with the material?	Is the content organized correctly? Is the navigation unclear or difficult to understand? Is there a cue/clue/indication/hint/signal that tells you what to do or where to go? Does the overlapping of text (subtitle) and its audio commentary in an/the educational video is effective/helpful/good? Is the design/layout confusing and difficult to understand/comprehend? Is the font size suitable? Are the graphics (visuals) too complex? Does the narration and animation are redundant (i.e., unnecessarily repetitious) in any way?

### 3.2 Phase 2: Expert review

#### 3.2.1 Identification of experts

Once the instrument has be developed, it was examined by five experts, all of them are PhD-holding academics from Malaysian universities with the rank of Associate Professor. Three of the specialists are experts in human computer interaction (HCI), one in medical education, specifically on cognitive load, and another in applied linguistics (English language). The experts were asked to assess the content, the clarity of the instructions and questions, the format, the scale indication chosen, the organization and flow of the questions, grammar, and spelling. They were provided with a brief description of the project and the instrument development process flow as additional materials.

#### 3.2.2 Input from experts (offline and online)

The experts were given a period of time to evaluate the instrument developed, shown in Table 2. These evaluations were done asynchronously, and their evaluations were analyzed. Online sessions through MS Teams [66] were conducted to validate and verify the input provided to ensure that the information received is accurate. The input from the experts is categorized into content, structure and scale, and language is shown in Table 3. It also showed the changes that were implemented.

**Table 3:** Comments from the experts based on content, structure and scale, and language, and changes implemented in pretesting and pilot experiment.

Experts	Content	Structure and scale	Language (clarity, grammar)
Expert 1 (HCI)	No issue on content	Should use 4-point Likert scale	No issue on language
Expert 2 (HCI)	Change questions to sentences. Questions should be categorized into sections. Use first person sentences to make the instrument more “human”.	Maintain the 5-point Likert scale	Some of the sentences are ambiguous, need to be more specific
Expert 3 (HCI)	No issue on content	Suggest using either 4-point or 5-point Likert scale	Need refinement on some of the questions
Expert 4 (Cognitive Load)	Measurement should be done after each task. Therefore, instruction needs to be clearer and must relate directly to the task to ensure the answer is based on the learning experience. Change questions to sentences.	Suggest using either 4-point Likert scale or 10-point Semantic Differential scale	No issue on language
Expert 5 (English language)	Change questions to sentences	No issue on structure/scale	Questions with “or” or “/” should be separated to avoid confusion. Choose the correct and accurate word. Use words that are universally recognized as meaning the same thing.
Changes Implemented in Pretesting Experiment	Sentences have been categorized into sections, based on original criteria; content organization (CO), navigation (Nav), signaling/cue (SC), audio/video (AV), aesthetic and minimalist design (AM) and visual representations. Visual representations are further divided into two sections: font (Ft) and graphics complexity (GC). Some of the sentences was reversed to negative statements in order to avoid response bias.	Sentences will be using the 4-point Likert scale based on ipsative Likert scale. This is done to decrease the possibility of central tendency bias, which occurs when respondents take the convenient neutral stand where it does not contribute statistically to the analysis of the measures [67]	Questions have been changed to sentences. Ambiguous questions/statements have been modified and split into several statements. One of the main issues that has been identified by Expert 5 is the use of “graphics complexity” (refer to question no. 7 in Integrated questions based on constructs, in Table 2). In order to ensure the clarity of the word, the questions were split to number of objects, long menus, number of links, irrelevant images, colors used, and forms as explained by Refs. [[68], [69], [70], [71]].

### 3.3 Phase 3: Pre-testing and Phase 4: pilot experiment

#### 3.3.1 Identification of experimental details

Foundation and degree students, enrolled in computer science or information technology programs at Universiti Tenaga Nasional (UNITEN), Malaysia were invited to take part in this experiment. The Brighten [72] MLA has been chosen for the purposes of this study. Brighten is based on Moodle, a Learning Management System, that has been extensively customized for UNITEN's students. This app is available in both web and mobile versions. Students can utilize the Brighten program to download notes, upload assignments, answer quizzes, exchange messages, and participate in discussions. Participants were required to bring their own mobile phones, ensuring their familiarity of the general UI and functionality of their own devices.

#### 3.3.2 Experiment implementation

In order to understand the ECL faced by the participants due to the UID of the mobile learning app, there are three specific tasks involved in the experiment. These tasks are the common tasks implemented on mobile learning apps.

• Task 1- Find, download, and view notes.
• Task 2- Find and watch videos.
• Task 3- Answer quizzes.

All of the tasks were designed to assess content organization, navigation, signaling/cues, aesthetic and minimalist design, font, and graphics complexity, with one additional evaluation on audio/video for Task 2. There were two conditions introduced for every task, as shown in Table 4.

**Table 4:** Different conditions for each task.

Task	Condition 1	Condition 2
Task 1- Find, download, and view notes	Layout A is where the content of the subject is arranged based on chapters (refer Fig. 3 (a))	Layout B is where the content of the subject is arranged based on activities (refer Fig. 3 (b))
Task 2- Find and watch videos	Video with subtitles	Video without subtitles
Task 3- Answer quizzes	All questions in single page	Questions were presented in multiple pages based on the number of questions

Once the participants have evaluated the UID criteria, they are required to rate the NASA-TLX evaluation [73]. NASA-TLX score is used to see the result of overall cognitive load. The correlation results between the score and the evaluated UID criteria will be used to identify the UID criteria that relates to the ECL of the participants.

In order to understand the specific issues faced by the participants, they will be asked open-ended questions about the challenges they encounter when performing the task. They will also be asked how the efficiency and the design of the MLA can be improved to increase the usability of the system.

#### 3.3.3 Informed consent

All participants were informed about the procedure in detail, verbally and through written statements. They were required to sign an informed consent. Participants were aware that they could withdraw their data at any point in the study.

### 3.4 Phase 5: Data analysis

In pretesting experiment, the questionnaire was measured by implementing the 4-points Likert scale based on the suggestions from the experts and following the ipsative scale [67]. However, the pretesting experiment shows contradictory results between the questionnaire section with the open-ended questions section. Therefore, the scale has been changed to 10-points scale, where 1 represents strongly disagree and 10 for strongly agree during the pilot experiment implementation, following the scale used in Leppink et al. and Cierniak et al. instrument [[26], [27], [28]]. All negative questions values were first converted to positive for a normalized comparison.

The results of the questionnaire in the experiments were analyzed based on their descriptive statistics as well as their Cronbach's alpha value for their internal reliability [74]. Spearman correlations were chosen to identify the correlations that exist between data, as the data were non-monotonic data.

The NASA-TLX is assessed based on 10-point scales, where 1 represents very low and 10 represents very high for mental demand (MD), physical demand (PD), temporal demand (TD), effort (EF) and frustration (FR). The performance (OP) is measured based on scale where 1 represents failure and 10 represents perfect. The results were categorized based on low, medium, somewhat high, high and very high [75].

Data from the open-ended questions (based on task) were analyzed to support and ensure the accuracy of the data collected. Repetitive words/phrases such as “crowded”, or “long scrolling” were identified and the frequency of the occurrences of these words were counted.

## 4 Experiment implementations and results

The experiment was divided into two major activities: pretesting and the pilot experiment. According to Ruel et al. [76], pretesting experiment was conducted to ensure the validity of the survey instrument and the metrics included in it and assesses the feasibility and rationale of the research from beginning to end. Pilot experiment runs the full study from beginning to end with the aim of increasing the main study's chances of success and helping in understanding the instruments' and participants' problem areas.

### 4.1 Pretesting experiment (Phase 2)

#### 4.1.1 Implementation

The experiments were conducted in a lab with a capacity of 30 people, in 8 different sessions. For each session, four to seven participants were involved. This is to ensure that the participants are comfortable and not overcrowded. The tasks will be done on their mobile phones, and they were asked to bring their headphones/earphones/earbuds if that is their normal practice when learning through their mobile phones.

**Fig. 4:**
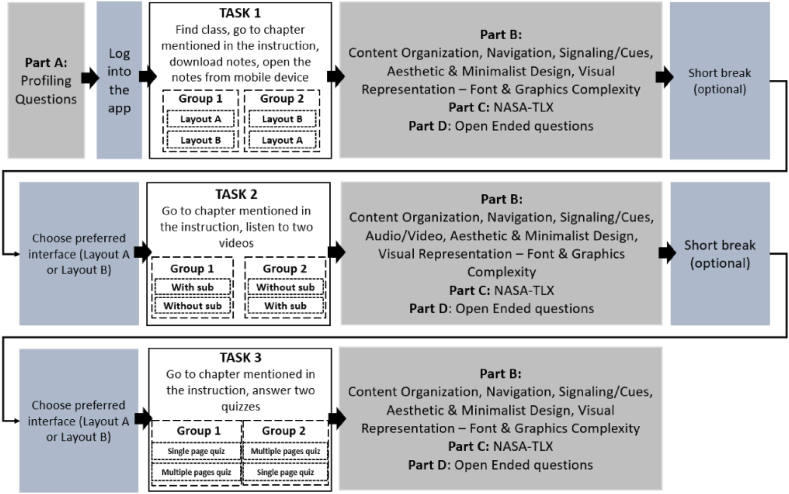
Experiment flow for pretesting experiment.

The participants were required to answer the developed instrument, NASA-TLX ratings, and open-ended questions, after they had completed each task. They will be answering the questionnaire using desktop computers through forms created using MS Forms [77]. The experiment flow is shown in Fig. 4. In order to ensure that the participants did not feel tired, they could choose to have a short break in between tasks.

The experiment adapts the within-subject method [78] where each participant will be doing all tasks, testing all conditions available:

• Task 1- Find, download, and view notes through both layout type
• Task 2- Find and watch two different videos (one with subtitles and another one without subtitles).
• Task 3- Answer quizzes with two different types of layouts

#### 4.1.2 Results from pretesting experiment

##### 4.1.2.1 Part A: Profiling

For the pretesting experiment, a total number of 39 participants were involved. 97 % of the participants have been using MLA for at least 1 year, with some of the participants having been using MLA for more than 3 years. However, only one-third of the participants felt that learning through mobile devices is easier compared to desktop PC.

Six of the participants' results were excluded from the analysis of the study, as they were not able to complete any of the activities in the experiment.

##### 4.1.2.2 Part B: Questionnaire and Part C: NASA-TLX

The reliability of the measurement depended on tasks. For each task, the internal consistencies were analyzed based on their UID criteria as shown in Table 5. All of the values are acceptable and sufficient as they are above 0.45 [74]. Cronbach's alpha with values that are 0.71 are categorized as good and relatively high. The results descriptive statistics were also shown in Table 5.

**Table 5:** Descriptive statistics and Cronbach's alpha values for each UID criteria.

UID Criteria	Task 1						Task 2						Task 3					
	α	μ	Md	Mo	σ	σ^2^	α	μ	Md	Mo	σ	σ^2^	α	μ	Md	Mo	σ	σ^2^
CO	0.535	2.765	3	3	0.928	0.862	0.674	3.140	3	3	0.760	0.578	0.743	3.144	3	3	0.780	0.608
Nav	0.825	2.924	3	3	0.884	0.782	0.697	3.235	3	3	0.737	0.543	0.736	3.212	3	3	0.697	0.485
SC	0.890	3.131	3	3	0.720	0.519	0.831	3.283	3	3	0.587	0.344	0.882	3.323	3	3	0.600	0.360
AM	0.633	2.924	3	3	0.611	0.373	0.779	3.167	3	3	0.592	0.356	0.858	3.288	3	3	0.622	0.387
Ft	0.813	3.309	3	3	0.568	0.323	0.821	3.217	3	3	0.631	0.399	0.854	3.285	3	3	0.591	0.349
GC	0.539	2.805	3	3	0.858	0.737	0.622	2.961	3	3	0.791	0.626	0.743	2.909	3	3	0.814	0.663

The descriptive statistics of the NASA-TLX results were shown in Table 6. The result of the interpretation of NASA-TLX scores show that for all three tasks, all the participants’ scores fall in the somewhat high to very high workload level, which shows high cognitive load (Table 7). It can also be seen that the number for participants that falls under the “high” and “very high” category work level were increasing as the task increases.

**Table 6:** Descriptive statistics of the NASA-TLX evaluation results.

NASA-TLX Evaluation	Task 1					Task 2					Task 3				
	μ	Md	Mo	σ	σ^2^	μ	Md	Mo	σ	σ^2^	μ	Md	Mo	σ	σ^2^
Mental Demand	3.394	3	3	2.561	6.558	3.152	3	3	2.612	6.820	3.727	4	3	2.388	5.705
Physical Demand	2.697	2	1	2.352	5.530	2.545	3	3	2.181	4.756	3.121	3	0	2.678	7.172
Temporal Demand	2.879	2	1	2.559	6.547	2.788	2	0	2.583	6.672	2.727	2	1	2.125	4.517
Performance	8.606	9	10	1.713	2.933	8.818	10	10	1.811	3.278	8.333	9	10	1.931	3.729
Effort	2.758	3	1	2.047	4.189	2.636	3	0	2.275	5.176	3.818	4	5	2.157	4.653
Frustration	2.303	2	0	2.352	5.530	2.242	2	0	2.180	4.752	2.545	2	0	2.306	5.318

**Table 7:** Number of participants based on the interpretation of NASA-TLX score.

NASA-TLX Score		Number of participants		
Workload Level	Score Range	Task 1	Task 2	Task 3
Low	0–9	0	0	0
Medium	10–29	0	0	0
Somewhat High	30–49	15	14	12
High	50–79	17	18	19
Very High	80–100	1	1	2

Despite the high number of participants with “high” and “very high” NASA-TLX scores, the only correlation that can be seen is between the NASA-TLX score and graphics complexity, for UID criteria.

##### 4.1.2.3 Part D: Open-ended questions

In pretesting experiment, the participants explained that when the layout is not according to their preferences, either by categories or by chapters, they identified the content to be unorganized. This leads to navigation issues. Ten of the participants reported that, initially, they failed to find the pre-enrolled courses, and they scrolled up and down several times, in order to find the course. When students log into the system, the top of their dashboard page displays the most recent courses they had accessed. As they have never accessed the pre-enrolled courses, they did not find the courses under “recently accessed course. They are required to scroll to bottom of the page to view all courses that they have enrolled in, which none of these participants did. Four of them were first-year students, with two in their first semester.

Another issue that the participants find disturbing is regarding the signaling/cue. Eight of the participants mentioned that they are not able to navigate and find the file to be downloaded easily because the font used for the chapter/activity title are almost similar to the other font.

11 of the participants mentioned that they are having problems understanding the video without the subtitles. Eighteen of the participants mentioned that they need to rewind the video without the subtitles several times. Twelve of the participants wore headphones/earphones during the session, however, six of them still need to rewind the video.

**Fig. 5:**
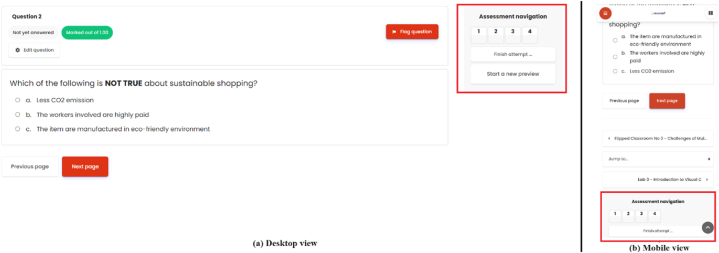
View comparison for the quiz with multiple pages between (a) desktop view and (b) mobile view.

Some other issues raised were the difficulty in navigating in between questions for the quiz with multiple pages. They compared the layout with desktop layout where they can easily jump from one question to another based on the navigation provided in the UID (refer to Fig. 5 (a)). Most of the participants failed to find the same navigation section on MLA as it was located at the end of the page (refer to Fig. 5 (b)).

##### 4.1.2.4 Issues

Pretesting experiments were designed for the participants to implement all the tasks from beginning until the end. It has been identified that there are several issues that arise. One of the main issues is fatigued and stressed. This is faced by most of the participants after doing the same process (implementing the task, answering the questionnaire, rating NASA-TLX and answering open-ended questions), repeatedly. This was casually mentioned by some of the participants after they had finished doing the final task. With this issue, there is an increased in the number of participants with “very high” NASA-TLX score, showing that emotions, particularly stress did affect the cognitive load of a learner [53,79,80].

Another issue would be the existence of contradictory answers between the item answered on questionnaire and the item answered in the open-ended questions sections. One example would be where a participant answered, “strongly agree” on the statement “I find that the structure of the mobile learning app is organized correctly”, but the participant also commented “the slides content are not organized properly in many of the pages I look”. To ensure the validity of the answer, several clarifications need to be made with the participants, post-experiments. Some of the participants mentioned that as the experiment is taking too long, they are not able to keep their focus on. Participants who experience mental fatigue may find it difficult to maintain their concentration on the job at task [81].

Some of the participants are not clear about the given task, and they did not get help from the facilitator, instead they continue trying to implement the tasks on different courses. Their data needs to be removed as they were not implementing the correct tasks on the correct course.

Another important argument identified in the pretesting experiment is that the correlation results do not reflect the answer provided in the open-ended questions and NASA-TLX score. This might be caused by the fact that the scale of the experiment is only 4 (Strongly Agree, Agree, Disagree and Strongly Disagree).

### 4.2 Pilot experiment (phase 5)

#### 4.2.1 Implementation

Based on the outcome of the pretesting experiment, some changes were implemented in the pilot experiment to ensure the precision and the validity of the results obtained. The changes were implemented to minimize the confusion faced by the participants when reading the statements.

The statements used in the questionnaires were re-evaluated and changed. One of the main changes were implemented in the content organization criteria section, the original statement, “I find that the heavy-content lecture materials are organized correctly”, was divided into three questions that were more understandable to the participants.

The evaluation scale was changed from 4-point to 10-point scale, as suggested by Expert 4, following the instrument designed by Leppink et al. [26,27] and Cierniak et al. [28]. The questionnaires which were originally presented on PC through MS Forms were changed to pen and paper, to minimize eye fatigue. As tiredness was the main issue identified in the pretesting experiment, the experiment method was changed from within-subject to between-subject. By doing this, the participants will evaluate less task(s). Task 1 was given to first trimester foundation students, as they were quite new with the UID of the app. Task 2 and task 3 were given to participants who are already familiar with the apps UID.

**Fig. 6:**
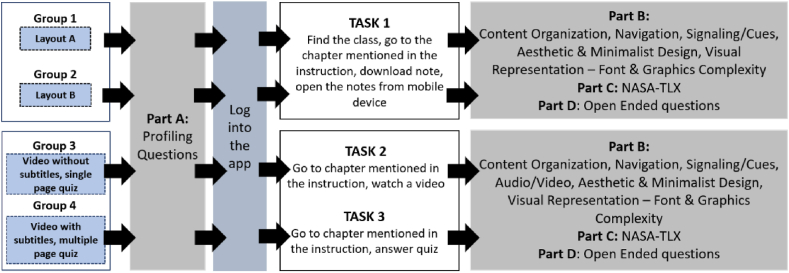
Experiment flow for pilot experiment.

Like pretesting experiment, participants were required to answer the developed instrument, NASA-TLX rating and open-ended questions, after they have completed the task. The experiment flow is as shown in Fig. 6.

#### 4.2.2 Results

##### 4.2.2.1 Part A: Profiling

For the pretesting experiment, a total number of 317 participants were involved. There are four groups of participants involved in the pilot experiment. For Task 1, students from Trimester 1, Foundation program were selected as they are very new in using the Brighten app. As for Task 2 and 3, participants were chosen from second- and third-year degree programs, where they are already very familiar with the Brighten interface. Despite the high number of participants involved in the pilot experiment, 33 of the data needed to be excluded as they were incomplete. The demographics detail of the participants, based on task, conditions, and operating system (OS) is as shown in Table 8.

**Table 8:** The demographics characteristics of the participants for pilot experiment.

Participants Information	Program of study	Task	Condition	Total number of participants	OS (based on accepted data)
Group 1	Foundation in Computer Science/Information Technology	Task 1 - Find, download, and view notes	Interface A (by chapters)	Total participants: 59 Data accepted: 51 Data rejected: 8	Android: 26 iOS: 25
Group 2			Interface B (by category)	Total participants: 81 Data accepted: 72 Data rejected: 9	Android: 36 iOS: 36
Group 3	Degree in Computer Science/Information Technology	Task 2 - Find and listen to a video and Task 3 - Answer quiz	Video without subtitles Quiz with all questions in a single page	Total participants: 103 Data accepted: 95 Data rejected: 8	Android: 41 iOS: 54
Group 4			Video with subtitles Quiz with questions in multiple pages	Total participants: 74 Data accepted: 66 Data rejected: 8	Android: 31 iOS: 35

##### 4.2.2.2 Part B: Questionnaire and Part C: NASA-TLX

The internal validity of the results can be seen as reliable as the Cronbach's alpha values were all more than 0.45. The internal validity and the descriptive statistics of the results are shown in Table 9. No data obtained on AV for Group 1 and Group 2 as their task were not related to audio and video. The descriptive statistics for the NASA-TLX results are shown in Table 10.

**Table 9:** The Cronbach alpha value and descriptive statistics for each UID criteria based on group.

UID Criteria	Group 1						Group 2					
	α	μ	Md	Mo	σ	σ2	α	μ	Md	Mo	σ	σ2
CO	0.798	6.993	7	10	2.506	6.281	0.694	7.133	8	8	2.223	4.943
Nav	0.889	7.284	8	8	2.309	5.331	0.806	7.438	8	8	1.999	4.010
SC	0.846	6.974	7	7	2.003	4.012	0.883	7.333	7	8	1.759	3.093
AM	0.816	6.294	7	7	2.407	5.796	0.685	6.924	7	8	1.972	3.890
Ft	0.934	7.945	8	10	1.966	3.864	0.950	8.244	8	8	1.455	2.12
GC	0.709	6.331	7	9	2.655	7.050	0.738	6.214	6	8	2.320	5.383
**UID Criteria**	**Group 3**						**Group 4**					
	**α**	**μ**	**Md**	**Mo**	**σ**	**σ2**	**α**	**μ**	**Md**	**Mo**	**σ**	**σ2**
CO	0.776	6.732	7	10	2.562	6.565	0.636	6.469	7	8	2.465	6.075
Nav	0.815	7.189	8	10	2.374	5.638	0.831	6.761	7	10	2.574	6.624
SC	0.921	7.112	7	9	2.467	6.086	0.830	7.019	7	8	2.280	5.198
AV	–	4.442	4	1	2.704	7.313	–	4.333	4	1	2.524	6.373
AM	0.909	5.926	6	7	2.506	6.279	0.668	5.558	6	7	2.236	5.000
Ft	0.965	8.112	8	10	1.781	3.173	0.937	7.629	8	8	2.036	4.146
GC	0.770	6.275	6	9	2.680	7.183	0.719	5.950	6	6	2.548	6.490

**Table 10:** Descriptive statistics of NASA-TLX results.

NASA-TLX Evaluation	Group 1					Group 2				
	μ	Md	Mo	σ	σ^2^	μ	Md	Mo	σ	σ^2^
Mental Demand	3.588	3	1	2.071	4.287	4.008	5	5	2.531	6.407
Physical Demand	3.314	3	1	2.267	5.140	4.403	5	5	2.430	5.906
Temporal Demand	3.294	3	1	2.081	4.332	4.444	5	5	2.489	6.194
Performance	8.686	9	10	1.643	2.700	8.556	9	10	1.555	2.419
Effort	3.882	3	1	2.769	7.666	4.806	5	5	2.453	6.018
Frustration	3.294	3	1	2.508	6.292	3.806	3	2	2.499	6.243
**NASA-TLX Evaluation**	**Group 3**					**Group 4**				
	**μ**	**Md**	**Mo**	**σ**	**σ2**	**μ**	**Md**	**Mo**	**σ**	**σ2**
Mental Demand	4.158	4	3	2.326	5.411	4.348	4	5	2.161	4.671
Physical Demand	3.474	3	1	2.396	5.741	3.734	4	4	2.041	4.166
Temporal Demand	4.053	4	1	2.294	5.263	4.014	4	5	2.304	5.309
Performance	8.379	9	10	1.739	3.025	8.507	9	10	1.771	3.136
Effort	4.189	4	1	2.655	7.049	4.130	4	5	2.268	5.145
Frustration	4.063	4	1	2.661	7.081	3.797	3	1	2.599	6.752

NASA-TLX interpretation scores show that for every group, there exist more than 65 % of the participants are having between high and very high scores of workload level; 67 % (Group 1), 75 % (Group 2), 73 % (Group 3) and 77 % (Group 4), as shown in Table 11.

**Table 11:** NASA-TLX interpretation of scores for every group.

NASA-TLX Score		Number of participants			
Workload Level	Score Range	Group 1	Group 2	Group 3	Group 4
Low	0–9	0	0	0	0
Medium	10–29	0	0	0	0
Somewhat High	30–49	17	18	26	15
High	50–79	32	44	57	45
Very High	80–100	2	10	12	6

The correlation coefficients were calculated using Spearman correlation coefficient as the associations between the variables were non-monotonic, which are the NASA-TLX score and the UID criteria. As the score for NASA-TLX were all between “somewhat high” to “very high”, the correlation coefficient results are seen as the UID factors that contributed to high reading of ECL through NASA-TLX score. The correlation coefficient results can be seen in Table 12 based on participant's group, tasks, and the OS of the mobile phone used. The correlation strengths were categorized according to the psychology field as weak (below 0.4), moderate (between 0.4 and 0.69), and strong (0.7 and above) based on the correlation coefficients obtained [82].

**Table 12:** Spearman correlation coefficient results.

Task/Group		Criteria	p-value (<0.05)	Spearman's rank correlation coefficient, rs	Interpretation of the correlation coefficient
Task 1 Overall		CO	0.01014	−0.231	Weak
		Ft	0.022	−0.206	Weak
		GC	0.003	−0.265	Weak
Group 1	Overall	No correlation identified			
	iOS	Ft	0.03	−0.434	Moderate
Group 2	Overall	CO	0.021	−0.272	Weak
		Ft	0.037	−0.246	Weak
		GC	0.004	−0.335	Weak
	Android	CO	0.0092	−0.4278	Moderate
		GC	0.0169	−0.396	Moderate
	iOS	GC	0.0316	−0.359	Moderate
Task 2 & Task 3 Overall		CO	0.000017	−0.329	Weak
		AV	0.0044	0.221	Weak
		GC	0.00217	−0.2377	Weak
Group 3	Overall	CO	0.00025	−0.367	Moderate
		GC	0.00126	−0.3261	Weak
	Android	CO	0.0035	−0.446	Moderate
		GC	0.0025	−0.460	Moderate
	iOS	CO	0.044	−0.276	Weak
Group 4	Overall	CO	0.034	−0.257	Weak
		SC	0.0132	−0.299	Weak
		AV	0.0011	0.387	Moderate
		Ft	0.0117	−0.304	Weak
	Android	CO	0.034	−0.382	Moderate
		SC	0.0039	−0.503	Moderate
		Ft	0.0252	−0.401	Moderate

All of the correlations were negative correlation except for correlations between NASA-TLX scores with audio/video criteria. Generally, for task 1, 2 and 3 weak correlations between NASA-TLX scores with some of the UID criteria were observed. These correlations become stronger once the groups were further divided based on the OS that the participants were using.

##### 4.2.2.3 Part D: Open-ended questions

In the open-ended section of the questionnaire, the participants mentioned the UID issues that affected them during the experiment. The issues were categorized based on the UID criteria that has been identified earlier and the number of participants that raised the same issue were calculated, as shown in Table 11. Most of the participants think that content organization and navigation is the main issue of the app.

Other non-related UID issues include slow response from the system, error displayed on page (in terms of codes), technical issue with the video and also page contents are not automatically refreshed.

##### 4.2.2.4 Issues

The only issue faced during the pilot experiment was the incomplete data. As the data were obtained through pen and paper method, there were some cases where the participants either missed or skipped some of the questions.

## 5 Discussions

The findings from the pretesting experiment aided in the development of a better instrument for measuring learners' ECL on the UID of a mobile learning application. They were able to provide insight into what was needed in the initial stages of instrument development. Some improvements have been implemented in the pilot experiments, based on these understandings. These improvements resulted in an improved result, with 14 weak correlations (correlation coefficient value less than 0.4), and 11 moderate correlation (coefficient value between 0.4 and 0.69). Most of the correlations found between the UID criterion in the instrument and NASA-TLX score findings were negative correlations. Negative correlations indicated that higher NASA-TLX scores are associated with more 'strongly disagree' responses in the ECL questionnaire. Although the correlation coefficient indicates weak or moderate relationships, the p-value suggests that the association is not due to random chance.

Tiredness was one of the most significant issues encountered when carrying out the pretesting experiment. All participants were required to implement a total of six tasks and answer the questionnaires three times. This leads to visual and mental fatigue. Visual or eye fatigue can decrease the performance of the participant [83]. Other than visual fatigue, repeated processes, and long experiment time were also among the contributing factors. These factors, causing mental fatigue, can increase participant errors, heighten boredom, and diminish motivation to continue the experiment [81,84]. In the pretesting phase, contradictory ratings were obtained from the same participant, where some giving high positive scores for questionnaire questions while stating that the same item is making the MLA inefficient in the open-ended questions. Another indication of weariness is that NASA-TLX scores increased as the task progressed. In this case, it cannot be proved that the higher score is due to the UID, as visual and mental fatigue may also contribute to the result.

Since the focus of this experiment is mental effort, it is critical to make sure that participant cognitive load comes from UID rather than fatigue or frustration. Using a PC to collect participant input merely increased their cognitive load because reading on a screen is more likely to cause fatigue than writing on paper [85]. Changing the experiment from within-subject to between-subject is one way to improve the execution of this research. This saves time and requires participants to just complete the task, once. Another way used was to fill out the questionnaire with pen and paper rather than using a desktop computer.

Despite expert assessments of the questionnaire, participants appeared to have trouble understanding the statements. This produced imprecise results in the pretesting experiment. Some of the original sentences were modified, and new sentences were introduced. As a result, in the pilot experiment, higher correlations were found since the participants were clearer about the item being rated.

In short, some of the suggestions that can be applied during the experiment include:

• Minimize eye fatigue. Use pen and paper to obtain input rather than PC/mobile device [85].
• Reduce the duration of the experiment. In a review paper [86], it was mentioned that experiment duration that are more than 30 min can produce mental fatigue.
• Minimize the task that the participants need to do. Limit each participant to only one/two tasks each. If the number of participants is low, divide the tasks to be done at different times. It is true that increasing the tasks can increase the reliability, but it can also increase fatigue and boredom on the participants [87].
• Ensure all items in the instrument are being evaluated. If using pen and paper for evaluation, remind the participants to ensure that they have evaluated all items.
• Ensure that the words used in the instrument were easy to understand for all participants. Other than endorsement from language experts, pretesting and pilot testing also helps in ensuring the targeted participants understand the statements/sentences used.

Following the pilot experiment result, it can be concluded that the content organization, graphic complexity, and font are the three primary factors contributing to the participants' ECL when using Brighten MLA (refer Table 12). It is evident from all three tasks, that these three UID criteria had an impact on the participants' ECL.

There was no relationship between the NASA-TLX score and the navigation criteria. Nonetheless, navigation was identified as another UID criteria that the learners find disturbing in the open-ended questions. The instrument did not capture issues such as “long scrolling”, “too much scrolling”, or “confusing navigation”. Modifications or new statements must be incorporated into the navigation criteria to gather more accurate data.

Signaling/cue was not identified as one of the ECL for group 3, however, this was also among the comments obtained in the open-ended section. It is proposed to include an additional line, “I can clearly differentiate sections/chapters/activities with the use of colors, highlights, or labels,” to ensure a more precise result. (see Table 13).

**Table 13:** Issues obtained from the open-ended questions.

Comments		Group 1	Group 2	Group 3	Group 4	Total Issues
CO	Difficult to find item	5	8	1	10	76
	UI too crowded/not organized	3	4	15	15	
	Too many categories/sub-categories		3			
	Too many items/objects/words	10		2		
Nav	Long scrolling	13	8	27	21	74
	Confusing navigation			5		
SC	Similar font color for every category/type of item (notes/quiz/assignment/etc.)			7		17
	No feedback after finish doing task/quiz			3		
	No idea what to do/where to go/lost			7		
AM	Different UI compared to web-based			4		4
AV	No subtitles			7		7
Ft	Font too small			2		2
GC	Cannot view the full image			2		2

From the results, it can be observed that out of six criteria evaluated, five of them contributed to the increase in the learners ECL. Some of the issues and possible explanation to these factors are shown in Table 14.

**Table 14:** ECL issues contributed by the UID of the MLA.

Factors	Issue (s)	Explanation and suggestions for solutions
Content Organization (CO)	Complexity - too crowded with too many categories or items	Information needs to be presented based on its relevancy and usefulness [35] to reduce complexity and increase productivity [35].
		Gestalt principles can be implemented, specifically proximity and similarity principle on layout, spacing and graphics [35] to reduce the complexity of the information representation [88]. Gestalt principles can enhance information processing and understanding [35,88].
		Design the content to be in small units in the simplest format [89]. It was suggested that the content was put in point form [90].
		The information presented should be adjustable and adaptable to the screen size [91]
Font (Ft)	Small font size, dense text and small text in complex images	Sans-serif font should be used [35,89,92] for simplicity and less complicated display [93]
		Standard size font is suggested to be 14–22 points [35] for buttons and not smaller than 10 points for text icon [93]
		Option should be provided option for learner to choose their font size [36,91,94], font thickness [95] and font color [91]
Graphics Complexity (GC)	Small image size, too many image, cannot view full image	Element wise zoom interaction should be enabled [36]
		Decorative visual effects such as animation and fancy background should be avoided [36] to minimize graphics complexity
		Alt-text description to graphics can be added [89] to assist learner to understand the graphics
Navigation (Nav) and signaling/cue (SC)	Long scrolling, confusing navigation	Basic tasks should be done in three or less clicks [91]
		Ensure the visibility of the buttons [35], main menu and options [91]
		Clue and search-related feature must be provided [35]
		Clear and consistent way to go back to the home screen and easy access to help functions should be provided [89]

The results of the correlation coefficient are interpreted as the UID factors that contributed to the high reading of ECL through the NASA-TLX score, since all of the result scores ranged from “somewhat high” to “very high”, for both pretesting and pilot experiment. The NASA-TLX scores for Group 4 participants show a moderate correlation with the audio/video criteria, proving that watching a video with subtitles will increase the learner's cognitive load. This is consistent with Mayer's redundancy principle [23], which states that text should not be added to spoken language.

Some of the correlation coefficients that were identified in pilot experiment as weak. However, moderate correlations can be observed once the groups were further divided into the OS that the participants were using when implementing the task. Although there are no obvious differences between the UID of Brighten MLA between iOS and Android, it can be said that Android users are having higher cognitive load compared to iOS users. This result supports the experiment that has been implemented by Alshehri et al. [96], where it has been reported that iOS users were having lower cognitive load compared to Android users.

The modified instrument can be found in the Appendix. Changes and additional statements included based on the outcome of the pilot experiment were italicized to show differences.

## 6 Conclusion

ECL can be reduced by avoiding anything that is not important to the learning tasks, and it can be measured through subjective methods. It is critical to determine which UID criteria cause the ECL on learners, as this load can be minimized with good UID.

The purpose of this study is to develop and evaluate a subjective instrument that can be used to measure extraneous cognitive load caused by user interface design of a mobile learning application. The initial constructs identified (depicted in Table 1) were further subdivided based on the input that were obtained from both pretesting and pilot experiment for clarity and detailed analysis. For example, originally the construct was just graphics complexity, but after the pretesting it was further subdivided into object and link clutter, long menus, relevancy of the image and color (refer Appendix). The identification of the constructs required to evaluate the UID factors of an MLA, that increase the ECL of a learner has answered RQ1.

The pretesting also provided some valuable information on the instrument validity and pilot experiment showed a better flow that can be applied when implementing the study later.

The four suggestions for better experimental setup that are applicable to endure better results have been previously explained in the results section and should be used to evaluate the UID factors of an MLA, that increase the ECL of a mobile learner. This has addressed RQ2 of the study.

According to the findings of the pretesting and pilot experiment, five out of six factors clearly affect the learners' ECL, which are content organization, navigation, signaling/cue, font and graphics complexity. The increased in the learner's cognitive load, can increase the time [97], and with the increased of time, it can reduce the efficiency of the apps, which will eventually reduce the usability of the app. By understanding this problem, not only the designer of the app can change the appearance of the app, but the course owner can also organize the course in a better way to help make the learning process easier.

Among the limitations of this study is that it has only been implemented on participants with a computer science/information technology background and only in English. It would be interesting to see if the results change with participants from different backgrounds and if the questionnaire is implemented in multiple languages, as English is not the first language for most of the participants.

Another limitation of this research is the lack of a benchmark study to evaluate the robustness of the instrument. While the existing study measures extraneous cognitive load to assess the learning materials [12], it does not specifically focus on evaluating the user interface design. On the other hand, cognitive load measurement tools such as NASA-TLX and QUIS that are tailored for user interface design do not provide a comprehensive assessment of extraneous load [60]. This suggests a need for further research to develop and validate a more holistic approach to measuring cognitive load, one that can effectively capture both the learning content and the user interface design aspects in mobile application usability studies.

As future work, it is suggested to conduct a deeper study on how the operating system of the mobile learning application (iOS or Android) affects the correlation results. Additionally, exploring the correlation between ECL and the mental health of participants prior to the experiment is recommended. One way to measure the mental health of participants cost-effectively is through the Original Bender Gestalt Test Based on One Stage Deep Learning (OBGESS), a computer-aided psychological drawing test with high precision [98].

## CRediT authorship contribution statement

**Masyura Ahmad Faudzi:** Writing – review & editing, Writing – original draft, Visualization, Validation, Resources, Methodology, Investigation, Funding acquisition, Formal analysis, Data curation, Conceptualization. **Zaihisma Che Cob:** Validation, Supervision, Resources, Project administration, Methodology, Investigation, Funding acquisition, Formal analysis, Data curation, Conceptualization. **Masitah Ghazali:** Validation, Supervision, Resources, Project administration, Methodology, Investigation, Formal analysis, Data curation, Conceptualization. **Ridha Omar:** Writing – review & editing, Writing – original draft, Funding acquisition, Conceptualization. **Sharul Azim Sharudin:** Validation, Supervision, Resources, Project administration, Investigation, Funding acquisition, Data curation, Conceptualization.

## Declaration of competing interest

The authors declare that they have no known competing financial interests or personal relationships that could have appeared to influence the work reported in this paper.

## Figures and Tables

**Table:** 

Content Organization: How is the data organized? If it is organized well, you should have no problem in implementing your task PERFECTLY and in a SHORT PERIOD of time.										
Statement	Strongly Disagree					Strongly Agree				
	1	2	3	4	5	6	7	8	9	10
1. I find that the structure of the mobile learning app is organized correctly.										
2. I find incomplete/inconsistent information.										
3. I can see that the related items and information are being grouped and arranged together.										
4. I find pages with excessive number of words.										
5. I can see that the images are organized appropriately.										
6. I think the information displayed in the mobile learning app is not crowded.										
**Navigation:** How do you move around the Brighten? Do you need to scroll up and down many times before you can find the item that you need? If it is easy to navigate Brighten, then you should find the item in **MINIMAL SCROLLING**.										
Statement	Strongly Disagree					Strongly Agree				
	1	2	3	4	5	6	7	8	9	10
*7. For me, the navigation process is confusing.*										
8. For me, the provided links lead to the correct destination.										
9. I find that navigating the app is easy.										
*10. I think the scrolling is too long*.										
**Signaling/Cue:** Do you know what button/link to click when implementing your task? Do you know that a text is a link? Or do you just click and see where it goes? If it is clearly signed, then you would **NOT** accidently **CLICK** on text that are **NOT LINK**										
Statement	Strongly Disagree					Strongly Agree				
	1	2	3	4	5	6	7	8	9	10
11. There is a clue (such as color code, highlights and labels) that tells me what to do.										
12. There is a clue (such as color code, highlights and labels) that tell me where to go.										
*13. I can clearly differentiate sections/chapters/activities with the use of colors, highlights, or labels.*										
14. I can clearly see the system progress.										
**Aesthetic and Minimalist Design:** Is the user interface design easy on the eyes? Or is it irritating?										
Statement	Strongly Disagree					Strongly Agree				
	1	2	3	4	5	6	7	8	9	10
15. I find the layout of the mobile learning app is aesthetically pleasing.										
16. I find the layout of the mobile learning app is easy to understand.										
**Audio/Video**										
Statement	Strongly Disagree					Strongly Agree				
	1	2	3	4	5	6	7	8	9	10
*17. I prefer animation/video with narration only, compared to animation/video with narration and subtitles.*										
**Visual Representation - Font**										
Statement	Strongly Disagree					Strongly Agree				
	1	2	3	4	5	6	7	8	9	10
18. The font style is suitable for me to read.										
19. The font style is suitable for me to explore the necessary material.										
20. The font size is suitable for me to read.										
21. The font size is suitable for me to explore the necessary material.										
22. To me, reading the font of the app is easy.										
**Visual Representation – Graphics Complexity**										
Statement	Strongly Disagree					Strongly Agree				
	1	2	3	4	5	6	7	8	9	10
23. There are too many objects in a single page of the app, I need to scroll.										
24. I find that there are long menus with subcategories of links.										
25. I find that there are excessive number of links.										
26. I can see that there is/are irrelevant image/images in the pages.										
27. I can see that there are too many colors used for fonts, headlines, tables, menus and backgrounds										
28. I found form in the mobile learning app.										
29. To me, understanding the graphics in the app is easy.										

## Appendix

Proposed Questionnaire to Measure Learner's Extraneous Load on User Interface Design for Mobile Learning Apps.

Open-ended Questions.

1. What is the most disturbing issue that you identify when implementing the task?
2. In your opinion, what can be done to improve the efficiency (less time taken) of implementing the task?
3. What can be done to improve the user interface design of the mobile learning apps?
